# First Comprehensive Characterization of Phayre’s Leaf-Monkey (*Trachypithecus phayrei*) Karyotype

**DOI:** 10.3389/fgene.2022.841681

**Published:** 2022-03-11

**Authors:** Xiaobo Fan, Krit Pinthong, Edivaldo H. C. de Oliveira, Alongklod Tanomtong, Hongwei Chen, Anja Weise, Thomas Liehr

**Affiliations:** ^1^ Bioengineering School, Xuzhou University of Technology, Xuzhou, China; ^2^ Jena University Hospital, Friedrich Schiller University, Institute of Human Genetics, Jena, Germany; ^3^ Department of Biology Faculty of Science, Khon Kaen University, Khon Kaen, Thailand; ^4^ Faculdade de Ciências Naturais, ICEN, Universidade Federal do Pará, Campus Universitário do Guamá, Belém, Brazil

**Keywords:** chromosomal rearrangements, multicolor banding (MCB), *Trachypithecus phayrei* (TPH), evolutionary conserved breakpoint (ECBs), fragile sites

## Abstract

The chromosomal homologies of human (*Homo sapiens*—HSA) and *Trachypithecus phayrei* (TPH—Phayre’s leaf-monkey, family Cercopithecidae) have previously been studied by using classical chromosome staining/banding and fluorescence *in situ* hybridization (FISH) from the 1970s to 1990s. In this study, we carried out molecular cytogenetics applying human multicolor banding (MCB), locus-specific, and human heterochromatin-specific probes to establish the first detailed chromosomal map of TPH, which was not available until now. Accordingly, it was possible to precisely determine evolutionary-conserved breakpoints (ECBs) and the orientation of evolutionary-conserved segments compared to HSA. It could be shown that five chromosomes remained completely unchanged between these two species, and 16 chromosomes underwent only intrachromosomal changes. In addition, 50 ECBs that failed to be resolved in previous reports were exactly identified and characterized in this study. It could also be shown that 43.5% of TPH centromere positions were conserved and 56.5% were altered compared to HSA. Interestingly, 82% ECBs in TPH corresponded to human fragile sites. Overall, this study is an essential contribution to future studies and reviews on chromosomal evolution in Cercopithecidae.

## Introduction

*Trachypithecus phayrei* (TPH), also known as Phayre’s leaf monkey or Phayre’s lutung (Behie and Groves, 2016), belongs to old-world monkeys (OWMs), family Cercopithecidae, subfamily Colobinae—the latter including an African and an Asian clade. The genus *Trachypithecus* comprises 17 species with one Asian colobine—TPH (Pinthong et al., 2018). TPH is widely distributed in continental Southeast Asia including India, Bangladesh, Western Myanmar, Thailand, Laos, Vietnam, and Southern China (Muul, 2002). It is important to notice that genus TPH was initially denominated with different Latin names, such as *Semnopithecus phayrei* and *Presbytis phayrei*, before the current name came into use (Gupta and Kumar, 1994).

The pedigree and chromosomal evolution of Hominidae has been principally and roughly resolved in previous cytogenetic and molecular cytogenetic studies; however, some gaps remain, including the karyotype of TPH (Stanyon et al., 2008). The latter was first described in 1970 as 2*n* = 44 (Hsu and Benirschke, 1970). In 1981, G banding revealed for a male TPH the karyotype composition is as follows: 22 (M) + 18 (SM) + 2 (A), XX (SM) (Chen et al., 1981). In 1998, chromosomal homologies between human and TPH chromosomes were established by FISH applying human whole chromosome paintings. This revealed unique reciprocal translocations corresponding to chromosomes of (*Homo sapiens*) HSA 1 and 19, and HSA 6 and 16 as well as fusions of HSA 14 and 15 and HSA 21 and 22 (Nie et al., 1998). In 2018, the subspecies TPH *crepuscula* was studied by GTG-banding and NOR staining (Pinthong et al., 2018).

Accordingly, up to now, there have been few or neither really comprehensive nor high-resolution FISH-banding–based (Mrasek et al., 2001; Liehr and Claussen, 2002; Weise et al., 2008) comparative molecular cytogenetic reports on homologies between HSA and TPH chromosomes. Thus, here, the first detailed comparative chromosomal map of TPH compared to HSA is presented, established by MCB and complementary heterochromtin- and one locus-specific probe(s). Furthermore, the results obtained in TPH were compared to karyotypes of Macaques (such as *Macaca fascicularis* = MMU) (Fan et al., 2014) and Silvery lutung (*Trachypithecus cristatus* = TCR) (Xiaobo et al., 2013), which were studied by identical high-resolution molecular cytogenetic approaches. Additionally, the relationship of ECBs with human fragile sites was analyzed.

## Materials and Methods

### Cell Culture and Chromosomal Preparation

An immortalized lymphoblast cell line derived from male TPH (#KKU-THPm6) was provided by the Department of Biology Faculty of Science, Khon Kaen University, Thailand. The animal was caught for less than 30 min from wilderness, its species was determined, and blood was acquired. Afterward the animal was set free again. Ethical review and approval were waived for this study due to the use of only a cell line.

### Fluorescence *in situ* Hybridization

Chromosomes were prepared from B-lymphocytes of the cell line according to standard procedures. FISH was done as previously reported using 24 human chromosome-specific multicolor-banding probe sets for all chromosomes (Mrasek et al., 2001; Liehr and Claussen, 2002; Liehr et al., 2002; Weise et al., 2008). Also, single and two-color FISH techniques were performed for mapping of ECBs by one locus-specific probe for the NOR region and human heterochromatin-specific probes on a probe set described previously (Bucksch et al., 2012).

### Microscopic Evaluation

Images were captured using an Axioplan II microscope (Carl Zeiss Jena GmbH, Germany) equipped with six corresponding filter sets for multicolor-FISH evaluation (DAPI, FITC, TR, SO, Cy5, and DEAC). Image analysis was done using pseudocolor banding and fluorochrome profiles of the ISIS digital FISH-imaging system (MetaSystems Hard and Software GmbH, Altlussheim, Germany). At least, 10–20 metaphases were recorded and applied probe or probe set.

## Results

**FIGURE 1:**
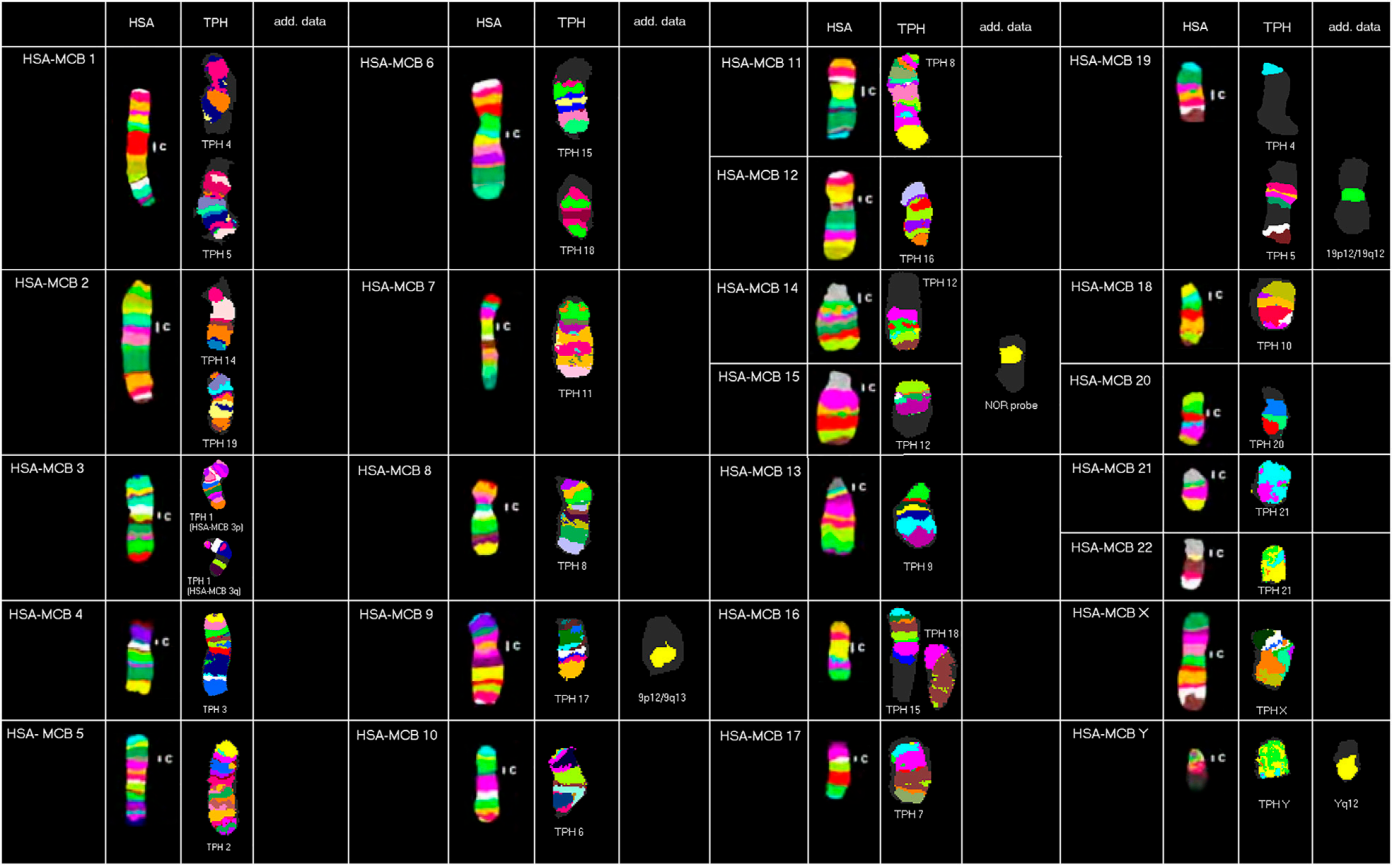
Representative results from this study using human MCB, NOR, and human heterochromatin-specific probes on TPH are depicted as pseudo-colored results for HSA and TPH (only valid for MCB results). The chromosomes are sorted here according to the HSA-chromosomes by using MCB. TPH chromosomes are numbered according to Nie et al. (1998).

Results obtained in molecular cytogenetic studies are summarized in Figure 1 and Table 1.

**TABLE 1:** Homologous regions, the centromere position (C), and colocalization with human fragile sites (FS). FSs are listed acc. to Mrasek et al. (2010).

Chr	Homologous to HSA chromosomes	Centromeric position	Fragile site
TPH1	5pter->5p14.1:5q11.2->5p14.1:5q21.1->5q11.2:5q35.3->5q21.1	as in HSA5	FRA5I, FRA5F, FRA5G
TPH2	3qter->3q28:3p23->3p24.3:3q22.1->3q25:3p23->3p21.3:3q28->3q25:3p21.3->3p12.3:3pter->3p24.3:3q22.1->3p12.3	neo 3q26	FRA3H, FRA3I, FRA3N, FRA3D, FRA3P
TPH3	4pter-4qter	neo 4q21.2	n.a
TPH4	19pter->19p13.11:1p22.2->1q22:1q43.2->1q22:1q43.2->1qter	as in HSA1	FRA1D
TPH5	19qter->19q13.2:1p33->1p22.2:19p13.11->19q13.2:1p33->1pter	as in HSA19	FRA1D
TPH6	10pter->10p11.23:10q21.1->10p11.23:10q21.1->10qter	as in HSA10	FRA10J, FRA10C
TPH7	17qter->17pter	as in HSA17	n.a
TPH8	11qter->11pter	as in HSA11	n.a
TPH9	13q11.1->13qter	neo 13q12.1	n.a
TPH10	18qter->18pter	neo 18q21.3	n.a
TPH11	:7p15.3->7q11.23:7p15.3->7p22:7q11.23->7qter	as in HSA7	FRA7J
TPH12	:15q11.1->15q26.3:C:14q11.1->14qter	neo 15q26.3/14q11.1	FRA15G, FRA15C, FRA14D
TPH13	8pter-8qter	neo 8p12	n.a
TPH14	:2q14.3-2qter	neo 2q24.3	n.a
TPH15	16qter->16p11.2:6q15->6pter	16p11.2	n.a
TPH16	12pter->12qter	as in HSA12	n.a
TPH17	9qter->9q22.32:9q12->9p34.3:9q12->9q22.32	neo 9q33.2	FRA9N
TPH18	16pter->16p11.2:6q22.31->6q25.3:6q22.31->6q15:6q25.3->6qter	neo 6q24.3	n.a
TPH19	:2q14.3-> 2q12.2:2p24.2-> 2q12.2:2p24.2-> 2pter	neo 2p14	FRA2T
TPH20	C:20q13.3-> 20pter	neo 20q13.3	n.a
TPH21	:21q11.1-> 21q22.3:C:22q11.1->22qter	neo 21q22.3/22q11.1	FRA21
TPHX	Xpter- > Xqter	as in HSA X	n.a
TPHY	Ypter- > Yqter	as in HSA Y	n.a

Overall, the majority of TPH chromosomes are completely homologous to one of the human chromosomes; exceptions are chromosomal exchanges that took place as follows: TPH 4 and 5 (homologous to HSA 1 and 19), TPH 12 (homologous to HSA 14 and 15), TPH 15 and 18 (homologous to HSA 6 and 16), and TPH 21 (homologous to HSA 21 and 22). The centromeric positions could be identified at the sub-band level for all 23 TPH chromosomes. In the following chromosomes, the TPH centromeric positions were the same as in HSA: TPH 2 (= HSA 5), TPH 4 (= HSA 1), TPH 5 (= HSA 19), TPH 6 (= HSA 10), TPH 7 (= HSA 17), TPH 8 (= HSA 11), TPH 11 (= HSA 7), TPH 16 (= HSA 12), TPH X (= HSA X), and TPH Y (= HSA Y). Centromere positions shifted compared to HSA as follows: TPH 1 (HSA 3q26), TPH 3 (HSA 4q21.2), TPH 9 (HSA 13q11.1), TPH 10 (HSA 18q21.32), TPH 12 (HSA 15q26.3/14q11.1), TPH 13 (HSA 8p12), TPH 14 (HSA 2q24.3), TPH 15 (HSA 16p11.2), TPH 17 (HSA 9q33.2), TPH 18 (HSA 6q24.3), TPH 19 (HSA 2p14), TPH 20 (HSA 20q13.3), and TPH 21 (HSA 21q22.3/22q11.1).

Furthermore, repetitive DNA was identified by human heterochromatin-specific probes as follows: the repetitive sequence D1Z5 located in HSA 1q11-q12 was not present in TPH 4 or TPH 5, while the region being present in human as the band 19p12/19q12 could be found in TPH 5. The human hemi-heterochromatic region 9p12/9q13 was located on the long arm of TPH 17, while D9Z3 (HSA 9q12) and D16Z3 (HSA 16q11.2) were not detectable in TPH. NOR signals can be found in the centromere region of TPH 21. Repetitive DNA in the human male in Yq12 also was observed in TPH Y. Overall, only HSA chromosomes 4 (TPH 3), 8 (TPH 13), 12 (TPH 16), X (TPH X), and Y (TPH Y) were completely unaltered during evolution between these two relatively distantly related species among OWMs.

Table 2 summarizes 50 ECBs observed in TPH in this study, which were identified according to the homologous regions in HSA. In addition, the characterized TPH breakpoints were compared with previously reported ones in TCR and in other macaque species using the MCB approach (Table 2).

**TABLE 2:** Colocalization of ECBs and FSs in TPH, TCR and Macaque species. Nomenclature and data acc. to (Xiaobo et al., 2013; Fan et al., 2014; Mrasek et al., 2010).

HSA chr	ECBs including neo-centromere in TPH	ECBs in TCR	ECBs in macaques	Fragile sites
1	1p33			n.a
	1p22	1p22		FRA1D
	1q22	1q22		n.a
			1q23.3	FRA1P
		1q24		n.a
		1q41		FRA1R
			1q42.13	FRA1H
	1q43.2			FRA1S
2		2p25.3		FRA2M
	2p24.2			FRA2C
	2p14			FRA2Q
			2p11.2	FRA2L
			2q11.1	FRA2R
	2q12.2			n.a
	2q14.3	2q14.1	2q14.1	FRA2
		2q21	2q21.1	FRA2F
			2q22.1	n.a
	2q24.3	2q24.2		FRA2T
		2q31		FRA2G
3	3p26.3	3p26.3	3p26.3	FRA3E
		3p25		FRA3F
	3p24.3		3p24	FRA3A
	3p23	3p23		n.a
			3p22.3	FRA3G
	3p21.3	3p21.3		FRA3H
	3p12.3		3p12.3	FRA3I
	3q22.1	3q22	3q22.1	FRA3N
	3q25	3q25		FRA3D
	3q26	3q26	3q26.1	FRA3O
			3q27.3	FRA3C
	3q28	3q28		FRA3P
4			4p15.3	FRA4D
		4p12		FRA4H
			4q10	n.a
	4q21.2			FRA4I
		4q22		FRA4F
5		5p15.2		FRA5H
	5p14.1			FRA5E
	5q11.2	5q11.2		FRA5I
	5q21.1	5q21		FRA5F
		5q31.2		FRA5C
	5q35.3	5q35.3		FRA5G
6		6p25.3		n.a
			6p24	n.a
		6p21		FRA6H
	6q15	6q15		FRA6G
			6q25.2	n.a
		6q21	6q21	FRA6F
	6q22.31			FRA6K
	6q24.3		6q24.3	n.a
	6q25.3		6q25.2	FRA6M
7	7p22	7p22.3	7p22.3	FRA7B
			7p22.1	n.a
			7p21.3	FRA7L
	7p15.3	7p15.3		n.a
		7q11.1		FRA7A
	7q11.23		7q11.23	FRA7J
			7q21.3	n.a
			7q22.1	FRA7F
8	8p12			n.a
9	9q34.3	9p34.2		n.a
		9q24.3	9p24.3	FRA9H
	9q12			FRA9F
			9q21.11	FRA9D
	9q22.32		9q22.33	n.a
	9q33.2	9q33	9q33.2	FRA9M
	9q34.3		9q34	FRA9N
10		10p15.3		FRA10H
	10p11.23	10p11.2	10p11.23	FRA10J
		10p11.1		n.a
			10q22.3	n.a
		10q11.1		FRA10G
	10q21.1	10q21.1		FRA10C
		10q22.3		FRA10D
11		11p15.4	11p15.4	FRA11J
		11q12		n.a
			11q13.4	FRA11E
12		12p13.33		FRA12F
13	13q12.1	13q12.1		n.a
			13q21.31	n.a
		13q14		FRA13G
		13q32		FRA13D
14	14q11.1	14q11.2	14q11.2	FRA14D
15	15q11.1-	15q11.2		FRA15C
			15q25	FRA15F
	15q26.3	15q26.2	15q26.3	FRA15G
16		16p13.1		FRA16H
	16p11.2			FRA16F
			16q22.1	FRA16C
			16q22.3	n.a
17		17p11.1		FRA17C
			17q12	n.a
		17q21.3	17q21.32	FRA17D
			17q23.3	n.a
			17q24	FRA17E
18	18q21.3	18q21	18q21.2	FRA18B
19		19p13.2		FRA19B
	19p13.11			FRA19B
	19q13.2	19q13.2		FRA19A
		19q13.43		FRA19A
20		20p12		FRA20B
			20p13	FRA20C
		20p11.1	20p11.21	n.a
		20q11.1	20q11.21	FRA20D
	20q13.3			FRA20
21	21q11.1	21q11.2	21q11.2	FRA21
	21q22.3			FRA21B
22			22p13	n.a
	22q11.1	22q11.21		n.a
X				
Y		Yp11.31		
		Yp11.2		
		Yq11.23		

**FIGURE 2:**
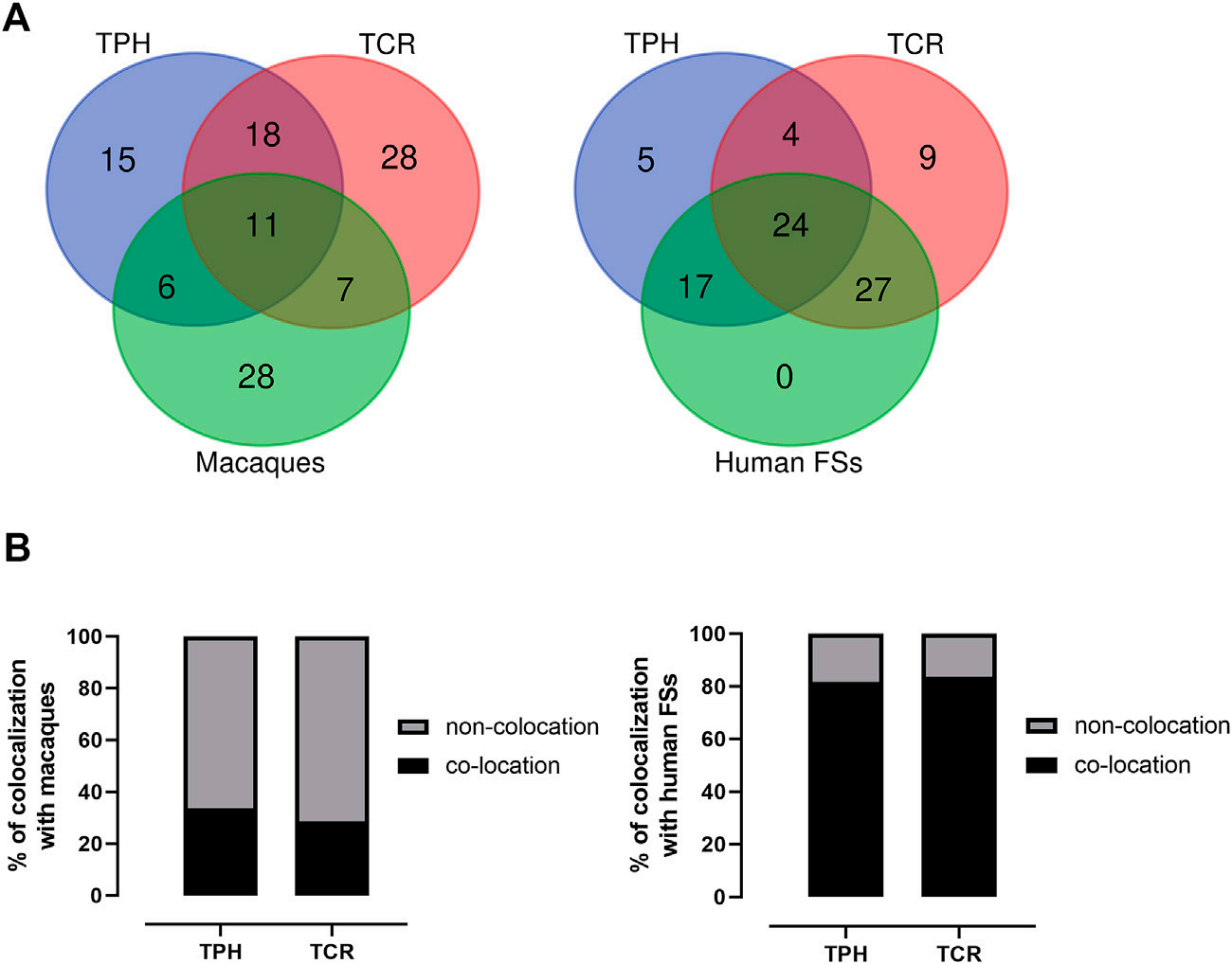
Identification of the relationship of ECBs in TPH with those in TCR, macaque species, and fragile sites. **(A)** Venn Diagrams depicting overlaps of TPH ECBs between TCR and macaques, and overlaps of the co-localization of ECBs in TPH with human fragile sites compared with the co-localization of ECBs in TCR with human fragile sites. **(B)** Left: quantification of the proportions of the co-localization of ECBs in TPH/TCR in macaques. In total, 32% of TPH ECBs and 26% of TCR ECBs were co-localizated in macaques. Right: quantification of the proportions of the co-localization of ECBs in TPH/TCR with human fragile sites. In total, 82% of TPH ECBs and 80% of TCR ECBs co-localized with human fragile sites.

The co-localization of ECBs among TPH, TCR, and in macaque species are listed with respect to HSA in Table 2. Out of 50 ECBs mapped in TPH, 29 (58%) and 18 (36%) coincided with ECBs in TCR and macaques, respectively (Figure 2; Table 3). Moreover, 41 (82%) reported ECBs in TPH co-localized with human fragile sites (Figure 2; Table 4).

**TABLE 3:** ECBs in TPH, TCR, and macaque species given as corresponding homologous human chromosome bands.

Species	Total	Human homologous bands
Macaques/TCR/TPH	11	2q14; 3p26.3; 3q22, 3q26; 7p22; 9q33; 10p11.2; 14q11; 15q26; 18q21; 21q11
TCR/TPH	18	1q22; 1p22; 2q24; 3p23; 3p21.3; 3q25; 3q28; 5q11.2; 5q21; 5q35.3; 6q15; 7p15.3; 9p34; 10q21.1; 13q12.1; 15q11; 19q13.2; 22q11
Macaques/TPH	6	3p12.3; 6q24.3; 6q25; 7q11.23; 9q22.3; 9q34
Macaques/TCR	7	2q21; 6q21; 10q22.3; 11p15.4; 17q21.3; 20p11; 20q11
TPH	15	1p33; 1q43.2; 2p24.2; 2p14; 2q12.2; 3p24.3; 4q21.2; 5p14.1; 6q22.31; 8p12; 9q12; 16p11.2; 19p13.11; 20q13.3; 21q22.3
TCR	28	1q24; 1q41; 2p25.3; 2q31; 3p25; 4p12; 4q22; 5p15.2; 5q31.2; 6p25.3; 6p21; 7q11.1; 9q24.3; 10p15.3; 10p11.1; 10q11.1; 11q12; 12p13.33; 13q32; 13q14; 16p13.1; 17p11.1; 19p13.2; 19q13.43; 20p12; Yp11.31; Yp11.2; Yq11.23
Macaques	28	1q42.13; 1q23.3; 2p11.2; 2q11.1; 2q22.1; 3p24; 3p22.3; 3q27.3; 4p15.3; 4q10; 6p24; 6q25.2; 7p22.1; 7p21.3; 7q22.1; 7q21.3; 9p24.3; 9q21.11; 11q13.4; 13q21.31; 15q25; 16q22.1; 16q22.3; 17q12; 17q23.3; 17q24; 20p13; 22p13

**TABLE 4:** ECBs in TPH and TCR colocalizing with human FSs.

Species	Total	Fragile sites/human homologous band
TCR and TPH FS co-localization	24	FRA1D; FRA2; FRA2T; FRA3D; FRA3E; FRA3H; FRA3N; FRA3O; FRA3P; FRA5F; FRA5G; FRA5I; FRA6G; FRA7B; FRA9M; FRA10C; FRA10J; FRA14D; FRA15C; FRA15G; FRA18B; FRA19A; FRA19B; FRA21
TCR FS co-localization	27	FRA1P; FRA1R; FRA2F; FRA2G; FRA2M; FRA3F; FRA4F; FRA4H; FRA5C; FRA5H; FRA6F; FRA6H; FRA7A; FRA9H; FRA10D; FRA10G; FRA10H; FRA11J; FRA12F; FRA13A; FRA13D; FRA13G; FRA16H; FRA17C; FRA17D; FRA20B; FRA20D
TPH FS co-localization	17	FRA1S; FRA2C; FRA2Q; FRA3A; FRA3I; FRA4I; FRA5E; FRA6K; FRA6M; FRA7J; FRA9F; FRA9K; FRA9M; FRA9N; FRA16F; FRA20; FRA21B
TCR and TPH no FSs at	4	13q12.1; 1q22; 3p23; 7p15.3
TPH no FSs at	5	1p33; 2q12.2; 6q24.3; 8p12; 22q11.1
TCR no FSs at	9	6p25.3; 9p34.2; 10p11.1; 11q12; 20p11.1; 22q11.21; Yp11.31; Yp11.2; Yq11.23

## Discussion

MCB combined with heterochromatin- and a locus-specific probe is suited best to characterize basic karyotypic features in primates, as shown in our previous studies (Mrasek et al., 2001; Fan et al., 2014; Fan et al., 2015; Xiaobo et al., 2013; Sangpakdee et al., 2018). In this study, the first comprehensive characterization of the karyotype of TPH was done; and a comparison with that in TCR and macaques was performed accordingly (Fan et al., 2014; Xiaobo et al., 2013). Our results confirmed and refined previous cytogenetic studies of TPH chromosomes, which were at a much lower resolution (Nie et al., 1998; Pinthong et al., 2018). These results extended to a detailed characterization of all TPH chromosomes aligned to HSA by MCB, that were not available before (Dutrillaux et al., 1979; Rhesus Macaque Genome Sequencing and Analysis Consortium et al., 2007). NOR was mapped to ECBs/fusion points of HSA 14 and HSA 15 (corresponding to TPH 12) confirming previous results (Pinthong et al., 2018). Compared to the basic Hominidea karyotype, five chromosomes remained unchanged in TPH, namely chromosomes 3, 13, 16, X, and Y, similar to those in TPH (Pinthong et al., 2018) and related species (Misceo et al., 2008). In addition, compared to HSA, complex chromosomal rearrangements (Table 1) first described here took place during the evolutionary process when the common ancestor of HSA and TPH diverged and may further continue.

ECBs must have undergone breaking and rejoining of double-strand breaks (Tsai and Lieber, 2010). These evolutionary conserved chromosomal changes could have been driven by several factors, such as the intrinsic instability of segmental duplications (SDs) enriched in the flanking regions of ECBs. SDs have been suggested to have a significant impact on genome plasticity during the evolution of primate chromosomes in previous studies (Kehrer-Sawatzki and Cooper, 2008). It is suggested that SDs within recombination hotspots might mediate non-allelic homologous recombination (NAHR). For example, two homologous SDs on the same chromosome, but in opposite orientation, could be the bases of an inversion. If SDs are in direct orientation, NAHR results in duplication and/or deletion as reported in human microdeletion-/microduplication syndromes and bases of copy-number variant regions (CNV’s) in human (Liehr, 2021). SDs located on different chromosomes can be the bases of NAHR-mediated chromosomal translocations (Tsai and Lieber, 2010; Gu et al., 2008).

While in previous reports, there were no detailed and characterized centromeric regions of TPH in corresponding reviews on OWMs (Ventura et al., 2004; Ventura et al., 2007; Stanyon et al., 2008), here, a first clue was possible about positions of centromeric regions in TPH (Table 1), that is, 56.5% TPH centromere positions shifted and 43.5% centromere positions were conserved compared to HSA. This is similar to the situation in TCR, that is, conserved centromeres in TPH kept their positions during evolution from common ancestors. However, these conserved centromeric regions normally do not have identical alphoid sequences as in HSA (Rocchi et al., 2012), and neo-centromeres are preferentially formed most often in gene deserts (Lomiento et al., 2008).

There are 29 identical ECBs in TPH and in TCR, and 17 ECBs are in concordance with those in macaque species. Moreover, 11 identical ECBs were identified in TPH, in TCR, and in macaque species (Tables 3 and 4). These findings are useful for the reconstruction of a common ancestral karyotype in further studies by applying, for example, locus-specific FISH-probes and/or sequencing of the TPH genome. In total, 41 (82%) of reported 50 ECBs in TPH corresponded to human fragile sites, which is in concordance to previous observations in TCR that ECB regions are highly connected to common FS locations (Francis, 2002, Mrasek et al., 2010; Fungtammasan et al., 2012). It has been suggested that FSs are low-stability regions, supporting their potential role in the formation of evolutionary chromosomal rearrangements (Mishmar et al., 1998). In this connection, others suggested the involvement of the cellular checkpoints proteins *ATR* and *BRCA1*, which are also critical for the expression of FSs (Casper et al., 2002; Arlt et al., 2006; Glover, 2006). Also, comparative analyses showed that the co-localization of ECBs in TPH/TCR with human FSs revealed no differences, indicating that Asian langurs are karyotypically closely related (Alekseyev and Pevzner, 2010).

In conclusion, the presented TPH karyotype and comparison to other langurs and macaques provided new insights into chromosomal evolution. It is another stepping stone in primate evolution research.

## Data Availability

Raw data supporting the conclusion of this article will be made available by the authors on request, without undue reservation.
